# Advances in materials and technologies for digital light processing 3D printing

**DOI:** 10.1186/s40580-024-00452-3

**Published:** 2024-11-04

**Authors:** Jisoo Nam, Miso Kim

**Affiliations:** 1 Department of Advanced Materials Science and Engineering, Sungkyunkwan University (SKKU), Suwon, 16419 Republic of Korea; 2 SKKU Institute of Energy Science and Technology (SIEST), Sungkyunkwan University (SKKU), Suwon, 16419 South Korea

**Keywords:** Digital light processing, 3D printing, Smart materials, Piezoelectric materials, 4D printing, Recyclable digital light processing, Multi-material digital light processing, Grayscale digital light processing, Artificial intelligence

## Abstract

Digital light processing (DLP) is a projection-based vat photopolymerization 3D printing technique that attracts increasing attention due to its high resolution and accuracy. The projection-based layer-by-layer deposition in DLP uses precise light control to cure photopolymer resin quickly, providing a smooth surface finish due to the uniform layer curing process. Additionally, the extensive material selection in DLP 3D printing, notably including existing photopolymerizable materials, presents a significant advantage compared with other 3D printing techniques with limited material choices. Studies in DLP can be categorized into two main domains: material-level and system-level innovation. Regarding material-level innovations, the development of photocurable resins with tailored rheological, photocuring, mechanical, and functional properties is crucial for expanding the application prospects of DLP technology. In this review, we comprehensively review the state-of-the-art advancements in DLP 3D printing, focusing on material innovations centered on functional materials, particularly various smart materials for 4D printing, in addition to piezoelectric ceramics and their composites with their applications in DLP. Additionally, we discuss the development of recyclable DLP resins to promote sustainable manufacturing practices. The state-of-the-art system-level innovations are also delineated, including recent progress in multi-materials DLP, grayscale DLP, AI-assisted DLP, and other related developments. We also highlight the current challenges and propose potential directions for future development. Exciting areas such as the creation of photocurable materials with stimuli-responsive functionality, ceramic DLP, recyclable DLP, and AI-enhanced DLP are still in their nascent stages. By exploring concepts like AI-assisted DLP recycling technology, the integration of these aspects can unlock significant opportunities for applications driven by DLP technology. Through this review, we aim to stimulate further interest and encourage active collaborations in advancing DLP resin materials and systems, fostering innovations in this dynamic field.

**Graphical abstract:**

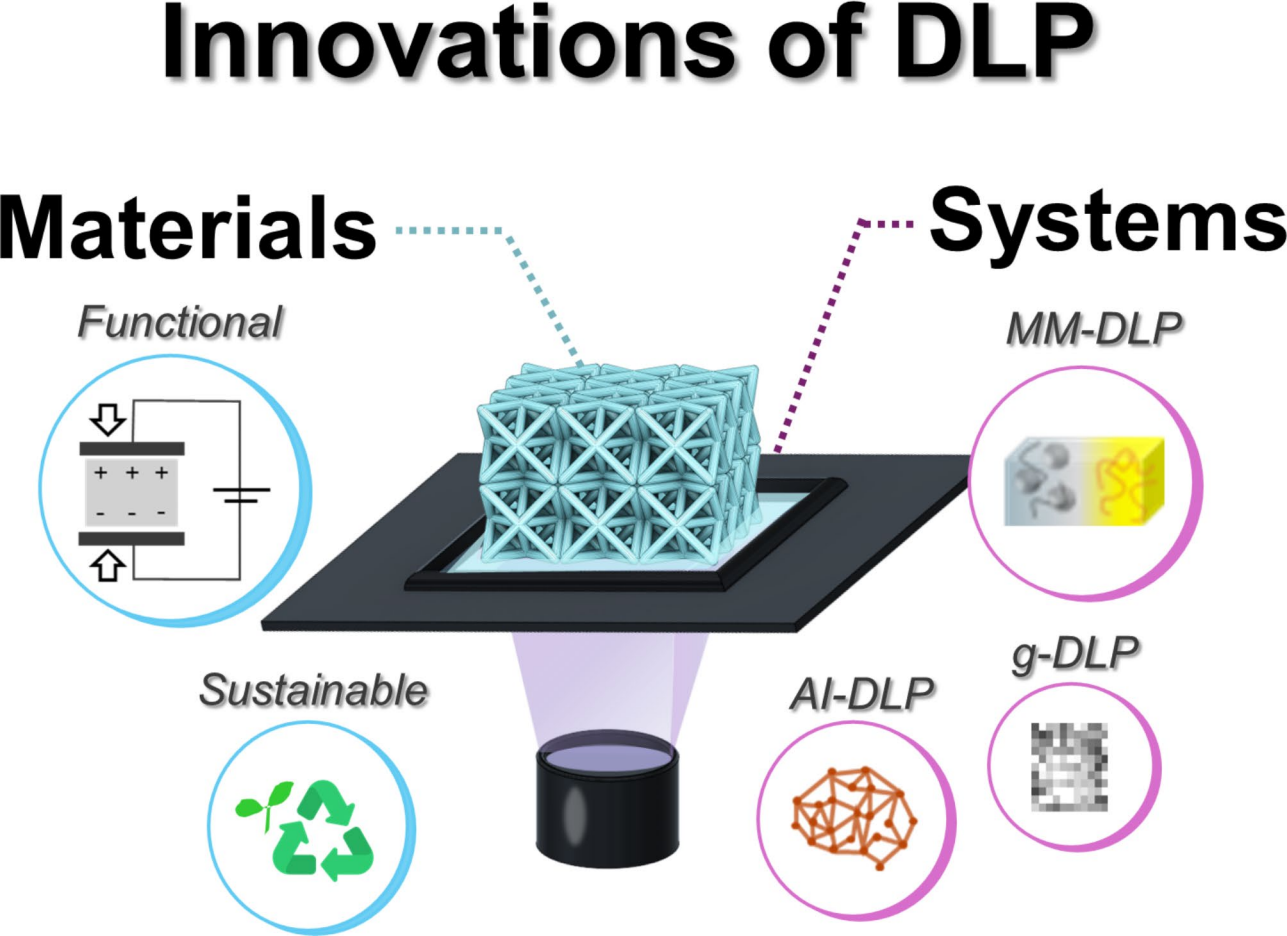

## Introduction

3D printing, also known as additive manufacturing, is a rapid prototyping technique originally developed for the accelerated production of prototypes. Over the years, it has evolved into a broad digital manufacturing technology [1]. This evolution has enabled the creation of complex structures that are impossible to produce using conventional manufacturing techniques, such as casting or machining, thereby opening new avenues for various applications. According to the ISO/ASTM 52939:2023 standard, 3D printing is classified into seven categories: powder bed fusion, material extrusion, material jetting, vat photopolymerization, binder jetting, sheet lamination, and direct energy deposition [2]. In vat photopolymerization, a light source is used to cure the liquid resin contained in the vat, leading to a photopolymerization process. Stereolithography (SLA), digital light processing (DLP), and liquid crystal display (LCD) are representative vat photopolymerization 3D printing technologies. SLA technology, being relatively early in development, uses a laser to cure liquid resin and offers high precision, but it has the limitation of slower printing speeds [3]. DLP utilizes the digital micromirror device (DMD), composed of a set of chipsets with distinct characteristics. Since the 1980s, DLP components, including DMD technology and sliced image-based 3D structure construction techniques, have been developed. In 1997, layer-by-layer printing using LCD technology was first proposed, and then in 1998, Laurence et al. attempted to use DMD instead of LCD to improve resolution, marking the beginning of modern DLP technology [4, 5]. The DMD allows for the projection of an entire layer at once, resulting in fast processing, high resolution, and smooth surface finishes [6, 7]. The DMD enables the simultaneous projection of an entire layer, leading to rapid processing, high resolution, and smooth surface finishes. However, the resolution in DLP printing is inherently linked to the number of pixels in the DMD projector, which imposes limitations on resolution beyond a certain threshold. To mitigate these issues, an LCD-DLP system has recently been proposed, utilizing an LCD screen as the light source for DLP 3D printing instead of redirected light projection [8, 9]. In this system, the maximum resolution is dictated by the pixel size on the LCD screen. While both DLP and LCD-DLP methods cure resin layer by layer, they differ in their light sources and processing techniques. Although the LCD-DLP method offers advantages in terms of resolution, the comparatively lower light intensity of LCD printing necessitates longer exposure times, which can diminish accuracy. Furthermore, the heat generated by the LCD may degrade or affect the properties of the photocurable resin. As a result, DMD-based DLP systems continue to be more commonly used. With advancements in both technologies, users can select the method that best aligns with their specific printing requirements.

Enhancing the interaction between light and resin materials and controlling the rheological properties within the vat is crucial to improving the accuracy of final products in DLP 3D printing. Studies in DLP have progressed from improving the rheological and photocuring properties of existing photocurable resins to developing stimuli-responsive and recyclable resins. Developing resins for vat photopolymerization with intrinsic functionality is challenging when incorporating materials such as conductive or ceramic fillers, which can hinder the light absorption required for proper photopolymerization. Overcoming these challenges involves precise control of photocuring parameters, such as light intensity and curing time, along with the optimization of resin formulations with additives like dispersants to achieve the ideal rheological and photocuring characteristics for DLP 3D printing. The development of environmentally friendly or recyclable photocurable resins and associated equipment is crucial to promoting sustainable manufacturing practices. While methods for recycling used photopolymerized products through pulverization or solvent dissolution have been proposed, a more comprehensive effort in refining resin materials and equipment is necessary for achieving true recyclability.

System-level innovations, including multi-material DLP, grayscale-DLP utilizing gradient light intensity, and artificial intelligence (AI)-integrated DLP, are actively being pursued. For example, multi-material DLP technology enables the fabrication of functional parts and conductive electrodes within a single structure to enhance device integration. Techniques utilizing gradient light intensity enable the 3D printing of programmable structures and enhance geometric precision. While the integration of AI in DLP 3D printing is still emerging compared with other 3D printing methods, promising advancements are being reported. Additionally, there are ongoing developments of portable and mass-production DLP equipment systems.

The remainder of this study is organized as follows: Sect. 2 discusses the principles of DLP, highlighting recent advances at materials and system levels. Section 3 discusses material innovations, including the development of smart materials in DLP, with a particular focus on DLP-enabled piezoelectric materials and applications as well as DLP-enabled 4D printing. The process of creating recyclable photocurable resins for circular manufacturing is also covered in Sect. 3. Section 4 discusses system-level innovations, exploring advancements in multi-material DLP, grayscale-DLP, the integration of AI with DLP, and the development of portable or mass-production DLP equipment. Finally, Sect. 5 addresses the challenges, future directions, and outlook for further advancements in this field.

## Fundamentals of DLP 3D printing

**Fig. 1:**
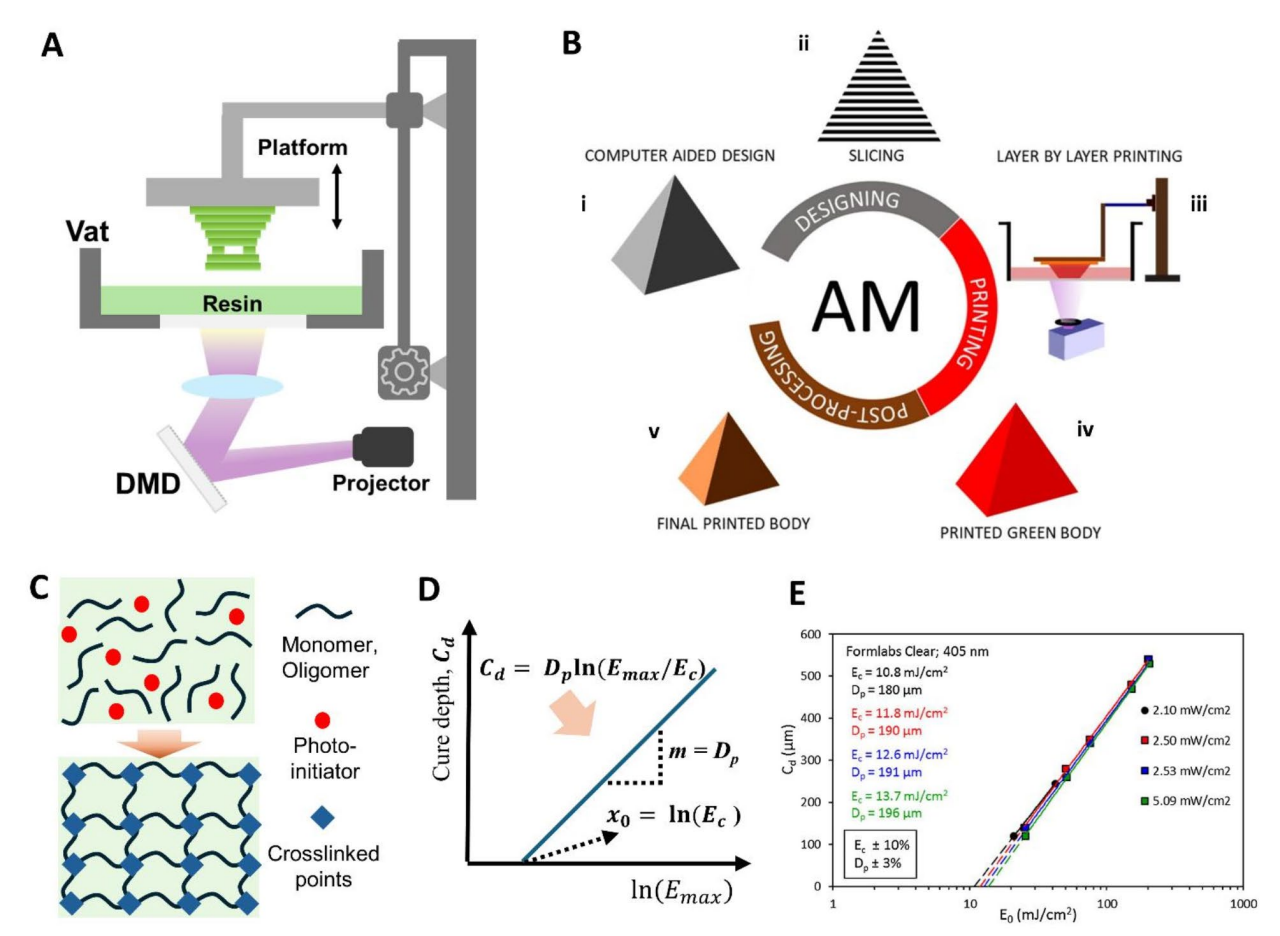
Fundamental principles of the DLP printing process and key components. (**a**) Main components of DLP equipment. (**b**) Step-by-Step process of DLP. Reproduced with permission from [12]. Copyright 2022, Springer Nature. (**c**) Schematic of the photopolymerization process. (**d**) Plotting Jacobs working curve and the relationship between the cure depth (*C*_*d*_), penetration depth (*D*_*p*_), light energy (*E*_*max*_), and critical energy (*E*_*c*_). (**e**) Examples for derived Jacobs working curve. Reproduced with permission from [20]. Copyright 2021, Elsevier

Figure 1a shows the main components of the DLP equipment, including a projector, a DMD, and a vat of photopolymer resin or suspension. Light is generated by a projector at the bottom, which passes through the DMD. DMD consists of arranged cells of microscopic mirrors, each corresponding to one pixel in the image. Thus, the light is illuminated to precisely follow the shape of the layer on the vat window. For DLP, users slice the 3D computer-aided design structure to be printed into thin layers, and the set of layer images is inserted into the printer. Each layer image works as a grayscale mask to selectively cure the resin (Fig. 1b). DLP 3D offers superior printing speed compared with the point-by-point curing method of SLA-type printers, owing to its plane-by-plane printing technique [10–12].

A photopolymer resin comprises photocurable or photosensitive oligomers and monomers in addition to a photoinitiator activated by a light with a specific wavelength and various chain extenders or crosslinking agents [13–16]. Figure 1c shows a simplified schematic of the photopolymerization process. The DLP parameters include light wavelength and intensity, exposure time, and layer thickness. Cure depth (*C*_*d*_), which is the cured thickness in one irradiance, is mediated by critical energy (*E*_*c*_) and penetration depth (*D*_*p*_), determined through the optical characteristics of the photocurable resin material, and light energy (*E*_*max*_), which is the energy of light emitted to the surface of the resins. The penetration depth (*D*_*p*_) is the 1/e penetration depth of light into the photopolymer, and the critical energy (*E*_*c*_) is the minimum energy required to initiate the photopolymerization. Based on the Beer-Lambert law, their relationship can be expressed as Jacobs equation (Eq. (1)), which describes the exponential decrease in light intensity as it penetrates a medium [17–19]:

1 $${C}_{d}={D}_{p}\text{ln}\left(\frac{{E}_{max}}{{E}_{c}}\right)$$

Experimental measurements can determine the Jacobs working curve of a given photocurable material, as shown in Fig. 1d. *D*_*p*_ is determined from the slope, and *E*_*c*_ is obtained from the x-intercept. Figure 1e shows the examples of graphs indicating how the slope and intercept are used to determine *D*_*p*_ and *E*_*c*_ in the Jacobs working curve [20]. The cure depth must be properly adjusted considering the target layer thickness. This helps prevent printing performance degradation, such as failure of interlayer adhesion or increased excess cure width (𝐶_w_), which is the additional cured area extending beyond the intended shape boundaries.

Various theoretical approaches have also been explored to improve the precision of printing results in DLP. For instance, a theoretical model has been developed to predict Jacob’s working curve, which relies solely on the absorbance characteristics of solid and liquid materials, as well as the gelation time of specific photocurable substances. In another approach, the curing behavior in DLP is modeled with greater sophistication by examining light propagation. However, aligning light exposure with the chemical reactions that drive curing presents significant challenges. Light propagation modeling can be conducted using one of two principal methods. The first method involves solving the complete set of coupled Maxwell’s equations; however, this approach is often computationally intensive and presents difficulties when simultaneously solving the chemical rate equations. Alternatively, the equations of radiative transfer can be employed, which considerably simplifies the modeling process by reducing the number of required equations from four coupled vector partial differential equations to a scalar integro-differential equation. For instance, Qi’s research group has integrated high-fidelity photopolymerization reaction kinetics into a reaction-diffusion physics-based model that considers the mass transport of free radicals [21]. The diffusion of these chemical species can blur the boundary between regions of dim and bright light, resulting in a loss of accuracy in the printing process. Their work has elucidated how processing parameters in photopolymerization—such as light exposure time and intensity—affect the overall cure state of a sample.

## Materials innovations in DLP 3D printing

### Functional materials and applications with DLP 3D printing

The integration of functional materials is essential for advancing DLP technology and broadening its applications across various industries. When combined with the unique architectures enabled by 3D printing, functional materials possessing diverse stimuli-responsive capabilities can significantly expand the range of applications through real-time changes in material properties and shapes [22–24]. Representative examples of functional materials include piezoelectric materials, shape memory alloys and polymers, thermochromic and electrochromic materials, magnetoelectric materials, self-healing materials, and hydrogels [25–30]. However, achieving the optimal conditions for vat photopolymerization while preserving the functionalities derived from these materials presents a considerable challenge. Consequently, numerous studies have focused on tailoring the composition and content of these materials, as well as optimizing process parameters, to ensure compatibility with DLP 3D printing while maintaining or enhancing the existing physical properties [25, 31, 32].

In this review, we focus on functional materials with a distinctive approach that sets our work apart from existing literature. Specifically, we delve into two key themes: first, the application of DLP 3D printing for piezoelectric materials and their associated applications (Sect. 3.1.1), and second, the integration of DLP 3D printing with smart materials in the context of 4D printing (Sect. 3.1.2).

#### DLP 3D printing for piezoelectric ceramics and composites

Piezoelectric materials possess the ability to generate an electric charge when subjected to mechanical stress, and conversely, they can undergo mechanical deformation in response to an applied electric field [33]. Consequently, these materials are extensively used in transducers, sensors, actuators, and energy-harvesting applications across biomedical and industrial sectors [34–46]. Piezoelectric materials can be classified into three primary categories: piezoelectric polymers, ceramics, and ceramic composites.

The 3D printing of piezoelectric polymers, particularly utilizing the well-known material polyvinylidene fluoride (PVDF) and its copolymers, can be achieved through a variety of techniques, including fused deposition modeling, direct ink writing, near-field electrospinning, and evaporation-assisted 3D printing [47–51]. However, there are few documented cases of directly photopolymerizing representative piezoelectric polymers like PVDF using stereolithography (SLA) or DLP 3D printing techniques. When it comes to PVDF-based piezoelectric nanocomposites, as detailed in the previous review article [52], applications have primarily been limited to inkjet printing and Fused Deposition Modeling (FDM).

The application of 3D printing technologies to piezoelectric ceramics and ceramic composites is also relatively limited. For example, there are numerous reports detailing the use of binder jetting for piezoelectric ceramics such as BaTiO_3_ and (K, Na)NbO_3_ (KNN) [53, 54]. Additionally, a growing body of literature documenting the use of SLA and DLP methods for BaTiO_3_ and other piezoelectric ceramic materials has emerged [55, 56]. Nevertheless, substantial challenges persist in the fabrication of ceramic slurries or suspensions, which complicate the 3D printing process [57]. This underscores the urgent need to enhance the accuracy of complex architectures, while further integration of alternative 3D printing techniques remains challenging. Moreover, the density and electrical properties—such as the dielectric constant and piezoelectric response-of 3D-printed piezoelectric ceramics are often inferior to those of ceramics produced by traditional non-3D printing methods. Overall, research on piezoelectric ceramics and the development of ceramic-based 3D printing technologies are still in their early stages compared to advancements in metal and polymer 3D printing. This highlights the critical need for further investigation and development in the field of 3D printing with piezoelectric materials.

The DLP process for piezoelectric ceramics or ceramic composites involves three main steps: ceramic suspension formulation, layer-by-layer curing through photopolymerization, and post-processing. The ceramic suspension is composed of ceramic particles and photocurable resins containing monomers and photoinitiators, dispersants, and other additives. The addition of ceramic particles leads to scattering of the absorbed light within the photocurable resin, which reduces the curing depth. Maintaining a high ceramic content is crucial to preserve the intrinsic physical properties of the ceramic [58–61]. However, increasing the ceramic particle content results in excessively high viscosity and poor flowability, making layer-by-layer printing challenging even with a doctor blade.

**Fig. 2:**
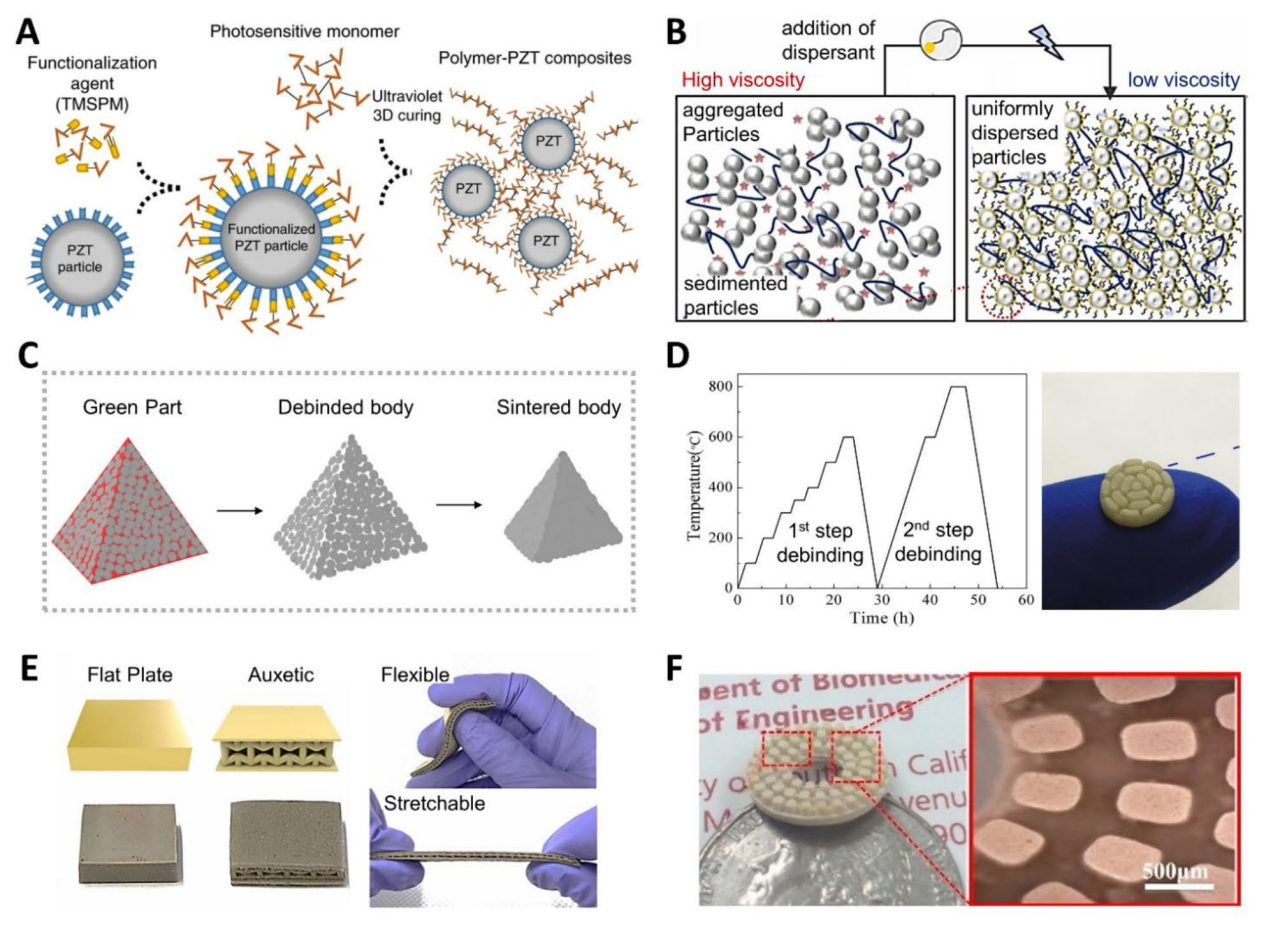
DLP 3D printing with piezoelectric materials and applications. (**a**) Surface functionalization of lead zirconate titanate (PZT) nanoparticles with TMSPM to enhance dispersion and piezoelectric performance. Reproduced with permission from [62]. Copyright 2021, Springer Nature. (**b**) Effect of dispersant on the stability and uniformity of PZT ceramic suspensions. Reproduced with permission from [64]. Copyright 2022, Elsevier. (**c**) Post-processing steps for 3D-printed ceramic structures, including debinding and sintering. Reproduced with permission from [12]. Copyright 2022, Springer Nature. (**d**) Two-step debinding temperature profile and optical image of the 3D-printed KNN green body. Reproduced with permission from [67]. Copyright 2019, Elsevier. (**e**) 3D-printed piezoelectric ceramic composite as a flexible sensor with an auxetic metastructure. Reproduced with permission from [63]. Copyright 2023, Elsevier. (**f**) Use of 3D-printed piezoelectric ceramic as a piezoelectric element for energy focusing and ultrasonic sensing. Reproduced with permission from [68]. Copyright 2016, Elsevier

To address these challenges, it is essential to lower the viscosity of the ceramic suspension while ensuring a stable and uniform dispersive state during the 3D printing process. This is achievable by surface-functionalizing the ceramic particles [62, 63] and optimizing the type and content of the dispersant [64]. For example, Cui et al. functionalized the surface of lead zirconate titanate (PZT) nanoparticles by introducing 3-(trimethoxysilyl)propyl methacrylate (TMSPM), as shown in Fig. 2a [62]. As illustrated in Fig. 2a, TMSPM is covalently grafted onto the PZT particle surface, and the strong covalent bonds between the piezoelectric nanoparticle and the polymer matrix improve the dispersion quality within the colloid, allowing the use of a high content of photocurable colloid resin. Furthermore, it has been experimentally shown that surface-functionalized PZT exhibits higher piezoelectric performance than without functionalization. This approach produced a high-resolution 3D-printed piezoelectric metamaterial composite architecture with arbitrary piezoelectric coefficient tensors and proposed vat photopolymerization-based 3D printing as a manufacturing route to implement it.

The type and content of the dispersant are also critical in controlling the rheological properties of the piezoelectric ceramic suspension and implementing high-resolution DLP 3D printing with enhanced surface quality [65, 66]. As shown in Fig. 2b, it is possible to maintain a uniformly dispersed high-loading PZT suspension when adding an appropriate amount of dispersant. However, an excessive amount of dispersant reduces the stability of the suspension, while an insufficient amount cannot achieve uniform dispersion. Studies have reported determining the optimal dispersant content for the viscosity of various ceramic suspensions, such as BaTiO_3_ and PZT. Furthermore, Kim et al. studied how the content of BYK-142, a dispersant, in the PZT-loaded ceramic suspension affected curing behavior, rheological properties, and suspension stability. It was found that the optimal dispersant content existed for high-resolution DLP 3D printing of PZT ceramics, along with curing parameters such as energy dose [64].

A critical aspect of formulating a ceramic suspension for DLP is the selection of an appropriate monomer. In the context of composites, the monomer serves as a polymer matrix after photopolymerization and plays a vital role in determining the final product’s mechanical flexibility and functionality, which must be aligned with the target application. For ceramics, since the photocured polymer component is subsequently removed from the final product, compatibility with the piezoelectric ceramic is essential. From this standpoint, minimizing the difference in refractive index between the ceramic and the monomer is advantageous, as it reduces light scattering and enhances light absorption, thereby improving curing depth. Additionally, it is beneficial for the monomer to possess low viscosity, especially considering that the viscosity of the suspension increases significantly upon the addition of ceramics. In this context, Liu et al. conducted a comparative analysis of the rheological and curing properties of four photosensitive monomers for DLP printing of BaTiO_3_ ceramics [69]. They identified acryloyl morpholine (ACMO) as the most favorable monomer due to its high refractive index among the tested options. Utilizing an ACMO-based resin matrix allowed for an increase in BaTiO_3_ content to 80 wt%, which likely contributed to achieving DLP 3D-printed BaTiO_3_ ceramics with significantly higher relative density and piezoelectric constants, even after the debinding and sintering processes. While substantial research has focused on various dispersants and monomer types in DLP studies involving BaTiO_3_, investigations involving other piezoelectric ceramics, such as PZT and KNN, are relatively few. Although some studies have employed a limited number of monomers for these materials, clear guidelines for determining the most suitable monomer for each specific piezoelectric ceramic remain ambiguous. As a result, DLP 3D printing of these materials tends to rely largely on empirical approaches. Therefore, further research is necessary to establish a more systematic understanding of the relationships between different monomers and specific piezoelectric ceramics.

After the structure is manufactured through DLP 3D printing, it becomes a green body as a ceramic composite. For composites, the process can be completed through straightforward post-processing steps, including rinsing, surface polishing, and, if necessary, post-curing. In contrast, creating a ceramic structure requires additional steps, including debinding to remove the polymer resin, followed by a sintering process for densification to obtain the final ceramic architecture, as shown in Fig. 2c [12]. One of the primary challenges of ceramic 3D printing technology is post-processing. During the debinding process, resin removal can create pores, undesirable defects, or even cause the structure to collapse. During sintering, shrinkage of the entire structure is unavoidable because the voids left by the removed resin are filled, and densification occurs between the ceramic particles at high temperatures. Therefore, the initial design must account for this shrinkage rate. Furthermore, compared with conventional ceramic molding processes that involve high-pressure pressing, the 3D-printed structure generally has a lower overall density. This lower density can negatively impact electrical properties, such as dielectric, ferroelectric, and piezoelectric characteristics. Therefore, various strategies are being explored to address these challenges, such as designing a multi-step debinding temperature profile with the slowest possible temperature change rate. Figure 2d depicts the temperature profile of a two-step debinding process for a 3D-printed green body of KNN, a lead-free piezoelectric ceramic, and the optical image of the resulting KNN structure [67].

The produced 3D-printed piezoelectric ceramic composite can be applied as a flexible tactile sensor with an auxetic metastructure shape (Fig. 2e) [63]. An optimal formulation of 3D-printable functionalized micro Pb[(Mg_1/3_Nb_2/3_)_0.1_Zr_0.45_Ti_0.45_]O_3_ (PMN–PZT) ceramic–polymer composites with a silane coupling agent and dispersant enabled enhanced surface quality, interfacial bonding, stable dispersion, and high piezoelectric properties of the final 3D-printed auxetic structure suitable for tactile sensors. Figure 2f demonstrates the use of 3D-printed piezoelectric BaTiO_3_ ceramic architectures in ultrasonic transducer applications for energy focusing and ultrasonic imaging [68]. 3D-printed piezoelectric structures are being utilized in various applications, such as ultrasonic transducers, sensors, actuators, and energy harvesting, realized through 3D printing technology [70].

#### DLP-based 4D printing of various smart materials

In DLP-based 4D printing, various smart materials have been introduced, including shape memory polymers (SMPs), shape memory polymer composites (SMPCs), liquid crystal elastomers (LCEs), and responsive hydrogels [71–73].

**Fig. 3:**
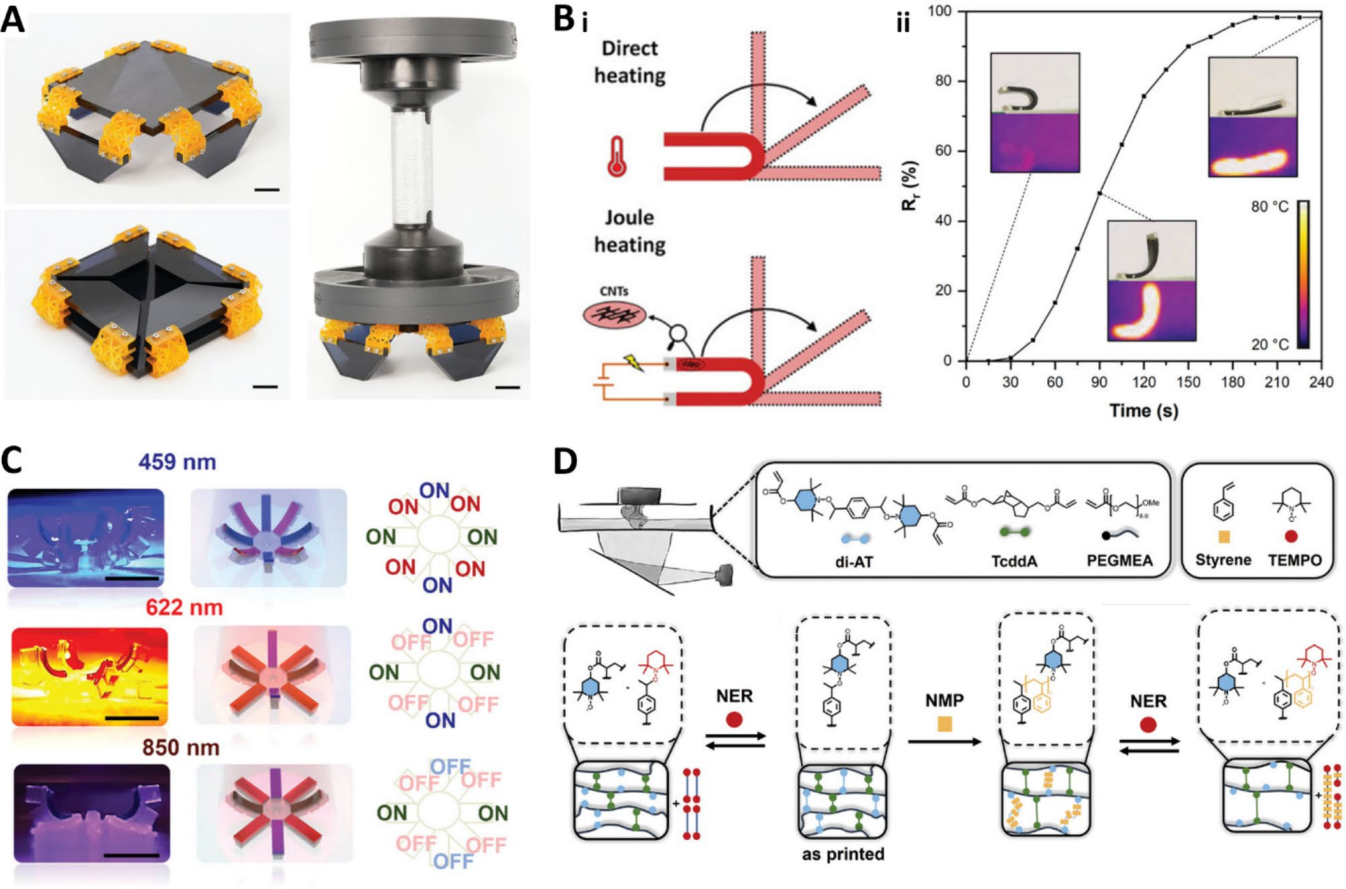
4D printing examples enabled by DLP 3D printing. (**a**) The smart table is programmed into a 2D compact shape, which can support heavy load with highly deformable SMP. Reproduced with permission from [76]. Copyright 2021, Wiley. (**b**) Remotely electro-activated shape recovery using tailored thermomechanical behaviors: (i) Shape recovery triggered by direct heating and Joule heating and (ii) variation of the Joule heating triggered shape recovery over time. Reproduced with permission from [80]. Copyright 2021, Wiley. (**c**) Octopus-inspired DLP-printed gripper-like multi-responsive actuator. Reproduced with permission from [83]. Copyright 2023, Wiley. (**d**) Schematic representation of ink formulation based on photo-cross-linkable alkoxyamine species; multi-way modification of printed structures via nitroxide-mediated polymerization (NMP) and nitroxide exchange reaction (NER). Reproduced with permission from [84]. Copyright 2024, Wiley

SMPs primarily employ thermal energy, transforming it into mechanical work. Compared to other soft active materials used in 4D printing [74], SMPs are distinguished by their higher stiffness and compatibility with a broad range of 3D printing technologies. For the manufacturing of advanced SMPs using DLP, mechanical robustness, UV-curability, reversibility, and a fast response with shape memory effects are required. Merckle et al. presented a method for synthesizing a thiol-ene photopolymerizable polyester resin tailored for DLP-based 4D printing, utilizing the well-established ring-opening copolymerization of anhydride and epoxide [75]. This resin exhibited robust mechanical properties, including an elastic modulus of over 600 MPa (with an elongation at break of 52.5%). In parallel, Zhang et al. developed a material characterized by its capacity for large deformations, fatigue resistance, and high-resolution printing. The developed SMP material demonstrated a substantial shape change of up to 1240% upon heating, and showed excellent fatigue resistance, withstanding over 10,000 cycles of repeated loading [76]. Figure 3a showcases the application of this material in the context of smart furniture. Although heat is the most widely used stimulus in SMP systems [77–79], there is a limitation that direct heat must be applied to induce shape change. In this regard, remote control becomes feasible by indirectly generating heat through other stimuli. Cortes et al. developed an electro-active SMPC by incorporating multi-walled carbon nanotubes (MW-CNT) into a matrix of polyethylene glycol diacrylate and poly(hydroxyethyl methacrylate) [80]. The integration of CNTs into the polymer matrix enabled electrical conductivity, facilitating shape deformation through Joule heating. This mechanism is depicted in Fig. 3b-i, with experimental results shown in Fig. 3b-ii. This approach demonstrates the potential for remote-controlled actuators. Similarly, Wang et al. introduced gold nanoparticles (AuNPs) into SMPs, utilizing them as photothermal converters to transform light into heat. The AuNPs absorbed light and elevated the SMP above its glass transition temperature (T_g_), thus enabling remote induction of shape change [81].

The research on DLP-based 4D printing has progressed to include a variety of external stimuli. While light is the predominant external stimulus utilized, the incorporation of light-responsive materials in DLP printing necessitates careful management due to potential interference with the photopolymerization process. Gastaldi et al. demonstrated the capability for high-resolution and precise light-responsive control in DLP 4D printing by employing an organic azobenzene dye [82]. The use of such organic dyes should be carefully managed, as problems such as slower curing speeds due to competition for absorption with photoinitiators can occur. However, it enables the realization of complex shape memory functions and allows for flexible deformation without the need for inorganic additives. Mainik et al. utilized a DLP 3D-printed bilayer structure composed of LCEs, where the LCEs served as active layers, and a non-responsive elastomer acted as a passive layer, facilitating controlled shape deformation [83]. Figure 3c shows an example of an actuator with multiple deformation modes, achieved by employing various organic dyes to create distinct absorption wavelength ranges across different regions. In other cases, Lantean et al. developed a magneto-responsive material using DLP, which responds to magnetic fields to alter its structure. This SMP exhibited various types of motion, such as rolling, translation, elongation, and folding/unfolding, making it suitable for applications requiring complex movements [84].

In addition to controlling shape deformation, active materials with variable mechanical properties represent a significant advancement in DLP-based 4D printing. Tran et al. introduced a method that utilizes dynamic chemistry to modulate the stiffness of the printed structures. Their approach incorporates alkoxyamine, which operates through a dissociation-combination mechanism involving nitroxide radicals. This mechanism is illustrated with chemical diagrams in Fig. 3d. This strategy enables precise control over reversible crosslinking and mechanical properties through nitroxide exchange reactions (NER) or nitroxide-mediated polymerization (NMP). In the NER pathway, a process referred to as “degrowing” is achieved by inducing the disconnection of dynamic bonds via the reversible homolysis of alkoxyamine in the presence of an excess of (2,2,6,6-tetramethylpiperidin-1-yl)oxyl. Conversely, in the NMP pathway, a “growing” effect is realized by immersing the printed structure in styrene overnight to allow for sufficient monomer diffusion, followed by polymerization at 125 °C under stirring conditions [70].

The primary strength of DLP technology lies in its remarkable potential to integrate a diverse range of material groups. Specifically, it facilitates the incorporation of both organic and inorganic materials into the photocurable resin matrix. In the realm of 4D printing, researchers have the opportunity to investigate various stimuli that can modify material properties. However, maintaining printability continues to pose significant challenges. For example, when adding inorganic materials, it is crucial to ensure adequate beam propagation within the composite matrix [73, 85]. Additionally, various organic materials must not disrupt the photopolymerization process [82]. Furthermore, as discussed in Sect. 4.2, the utilization of grayscale DLP technology allows for the integration of multiple properties within a single structure. This capability enables precise control over the shape of DLP-printed shape memory polymer (SMP) materials [77, 86]. This approach holds considerable promise for developing more sustainable and high-performance 4D-printed materials by reducing the energy required during the fabrication process [87, 88].

### Sustainability and environmental considerations for DLP 3D printing

**Fig. 4:**
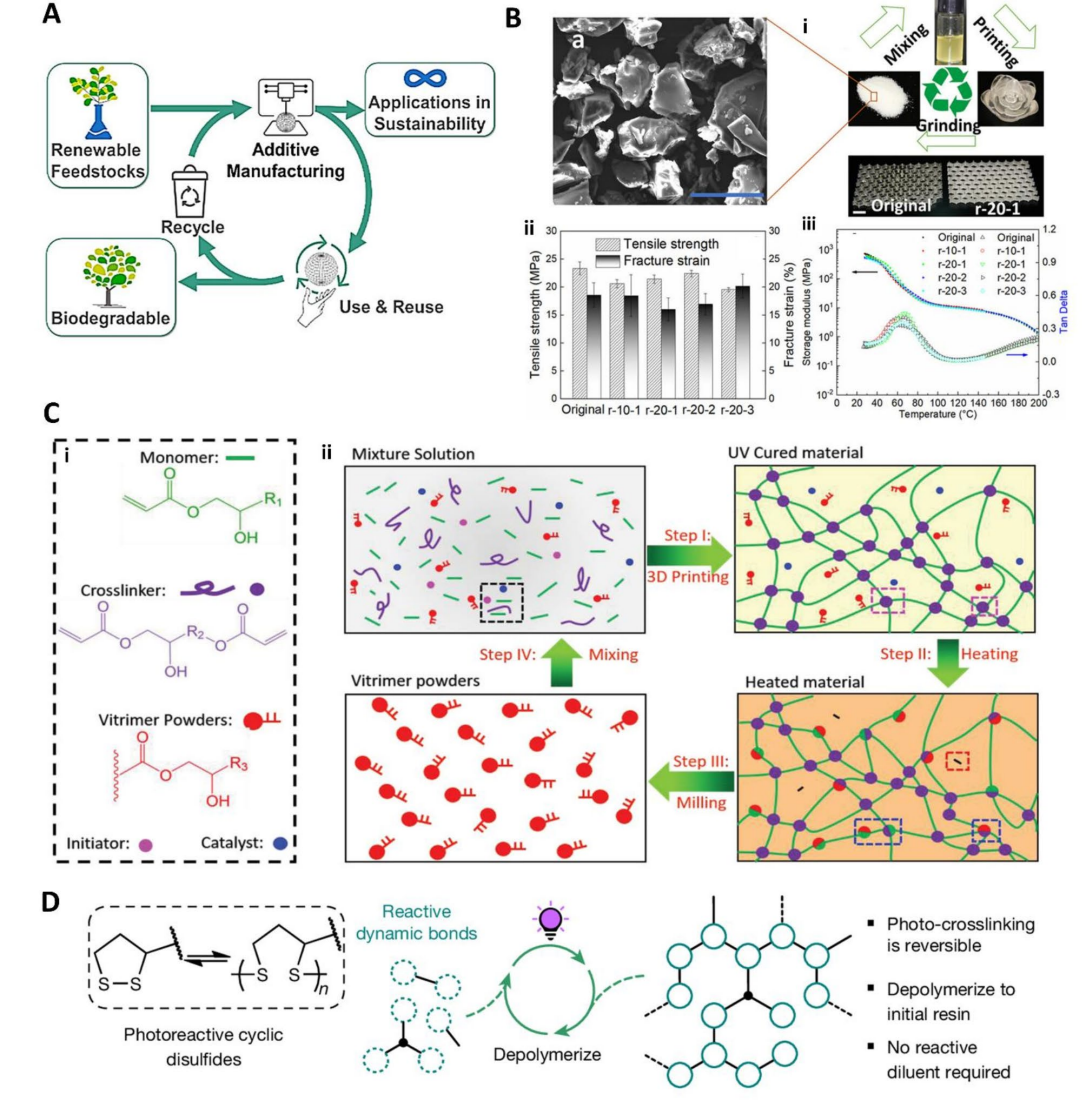
Recyclable DLP 3D printing. (**a**) Environmentally sustainable solutions for DLP 3D printing. Reproduced with permission from [91]. Copyright 2023, American Chemical Society. (**b**) The recycling loop of 3D-printed materials is shown in (i). Comparison of mechanical and thermal properties between original and recycled products, (ii) and (iii). Reproduced with permission from [93]. Copyright 2023, American Chemical Society. (**c**) (i) UV-curable solution formulation and (ii) process for recycling the transesterification-based vitrimer. Reproduced with permission from [94]. Copyright 2022, Wiley. (**d**) Light-mediated crosslinking and de-crosslinking utilizing photo-reactive strained cyclic disulfides, enabling a fully closed-loop recycling system. Reproduced with permission from [95]. Copyright 2024, Springer Nature

Sustainability and environmental considerations are crucial in additive manufacturing, including DLP 3D printing [89, 90]. Photopolymer acrylates, primarily derived from fossil fuels, have a significant carbon footprint. They usually create thermosets that are inherently non-recyclable and non-biodegradable. In DLP 3D printing, two major directions are considered for a more environmentally sustainable solution (Fig. 4a). The first approach is to efficiently recycle the 3D-printed products or resin remaining in the vat. The second solution is to develop renewable polymers based on bio-based materials with improved biodegradability [91].

Developing recyclable resin materials is essential for minimizing waste and reducing resource usage. The primary step towards circular DLP processing involves converting printed objects back into liquid resin. The dissolution of the linear polymer, isobornyl acrylate (IBOA), enables easy conversion of the printed objects into liquid toward depolymerization, which can be subsequently utilized for the same DLP re-printing. Consequently, this approach can be executed without complex chemical synthesis processes and applies to recyclable composites with fillers [92].

Dynamic covalent bonds can provide excellent recyclability for polymer systems using solvent- or heat-assisted strategies [96]. For instance, hydrogen bonding and ionic bonding with a dynamic nature in a photopolymer can be constructed from liquid resin consisting of urethane methacrylate, acrylic acid, and zinc dimethacrylate as the cross-linker [97]. Recycled samples are obtained through pulverization, heat treatment under a pressure of 10 MPa, and cooling, owing to the dynamic nature of the hydrogen and ionic bonds. Another example is hindered urea bonds to directly recycle solid polymers without any catalyst or solvent [93]. Figure 4b-i shows the recycling loop of the 3D-printed materials and honeycomb structures derived from the original resin and recycled resin denoted by r-x-y, where ‘y’ denotes the number of recycling times and ‘x’ indicates the wt% of the resin. Mechanical and thermal properties exhibit insignificant differences between the re-printed and original products, as shown in Fig. 4b-ii and 4b-iii.

Vitrimer is a polymer that combines the high mechanical and thermal properties and solvent resistance of thermosets with dynamic bonds to explore the reprocessibility of thermosets [93, 98]. The thermally activated bond-exchange reaction (BER) mechanism in vitrimers facilitates recyclability in DLP 3D printing. In a study by Li et al., a vitrimer was created using transesterification-based BERs between hydroxy and ester groups. Figure 4c-i and c-ii illustrate the recycling process of vitrimer through transesterification. A specially formulated UV-curable solution enabled the recycling of vitrimer under relatively mild conditions [94].

Despite advances in circular systems in other studies [96, 99], there is still a need to develop a fully circular, closed-loop recycling pathway. The closed-loop recycling resin can be depolymerized and directly reused without necessitating additional reactants. Recently, the Dove group developed a new photopolymer resin utilizing photo-reactive strained cyclic disulfides for a fully closed-loop recycling system, as shown in Fig. 4d [95]. This innovative approach enables light-mediated crosslinking and de-crosslinking and addresses the issues caused by orthogonality between the dynamic bonding formation and photopolymerization processes.

Likewise, researchers have explored the use of renewable materials in recycling systems derived from bio-based plant oils or other resources [100–102]. Additionally, vanillin methacrylate-functionalized Jeffamines offer an eco-friendly alternative to traditional petroleum-based polymers because they can be derived from natural resources. These materials also exhibit a wide range of Young’s modulus, including the characteristics typical of thermosets, making them suitable for diverse industrial applications and offering bio-sourced routes for vat photopolymerization resins [103].

## Recent innovations in DLP technologies

### DLP for multi-material 3D printing

In additive manufacturing, multi-material (MM) printing enables the creation of intricate composite materials with geometrically diverse properties, providing functionality, environmental adaptability, and improved mechanical characteristics [104, 105]. Among the various MM 3D printing technologies, MM-based DLP stands out for its compatibility with a wide range of materials, as well as its ability to produce high-resolution structures apace [106]. Additionally, it offers the advantage of excellent interfacial compatibility between heterogeneous resin materials [107]. However, MM-based DLP printing demands more sophisticated equipment compared to other printing methods, such as extrusion-type printers. This complexity is necessary to achieve precise material gradients while alternating between different liquid resins, in which the green body is repeatedly immersed. Therefore, this section explores the advanced equipment technologies, and the specialized materials developed for applications in MM-based DLP printing.

**Fig. 5:**
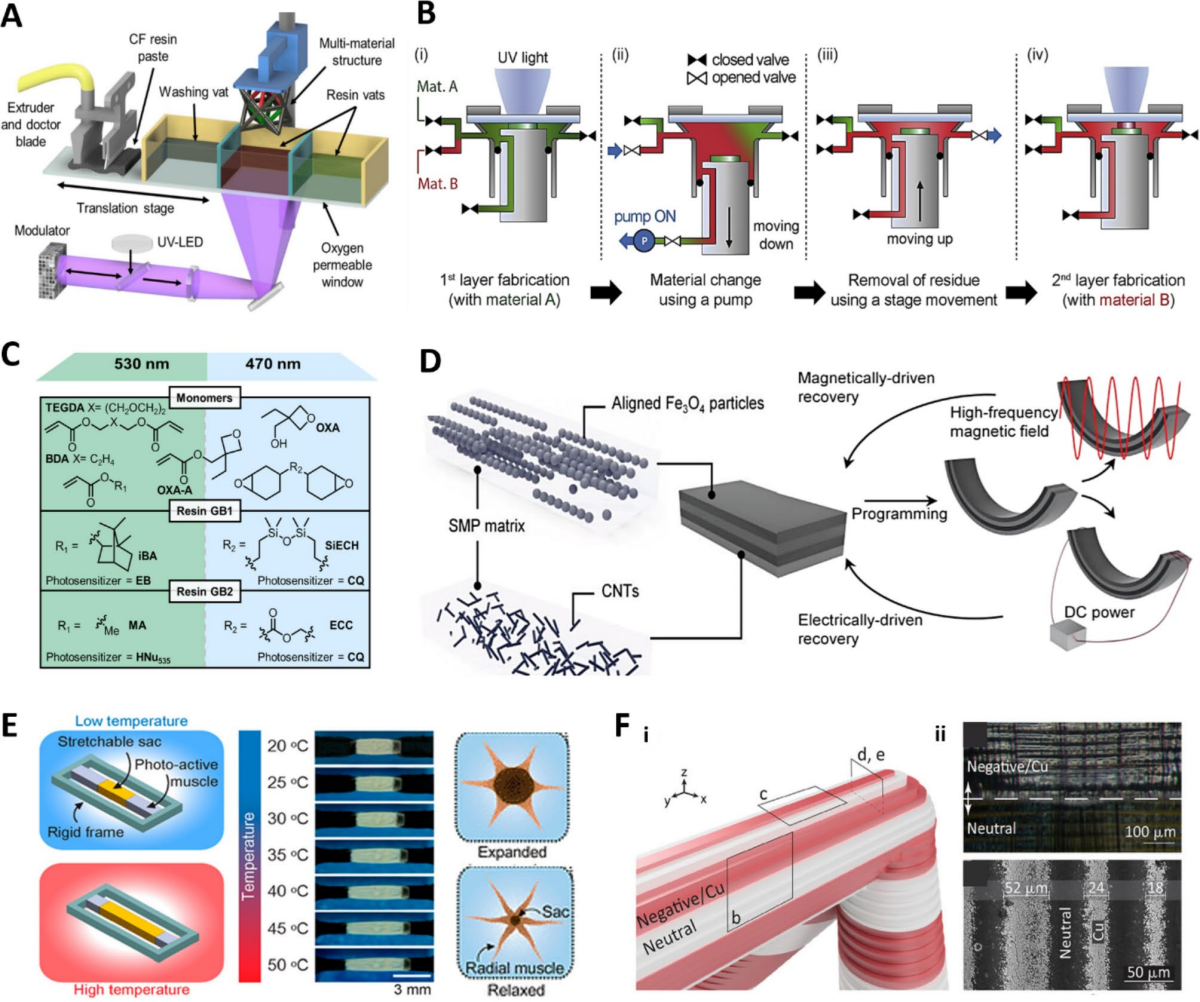
DLP technologies for MM 3D printing. MM-PµSL integrated with (**a**) a doctor blade of tape-casting process. Reproduced with permission from [108]. Copyright 2020, Elsevier. MM-PµSL integrated with (**b**) a dynamic fluidic control system. Reproduced with permission from [111]. Copyright 2019, Elsevier. (**c**) Schematic example of SMaLL technology. Reproduced with permission from [112]. Copyright 2021, American Chemical Society. (**d**) Dual-driven shape memory composites (SMC) and their functionalities. Reproduced with permission from [113]. Copyright 2024, Wiley. (**e**) Light-responsive artificial chromatophores (LACs) for color pattern modulation. Reproduced with permission from [114]. Copyright 2021, American Chemical Society. (**f**) Fabrication of (i) 3D-patterned conductive and dielectric architectures and (ii) selective Cu plating results. Reproduced with permission from [115]. Copyright 2024, Wiley

Various hardware features, such as resin change systems and multiple light sources, enable MM DLP printing. MM DLP technology usually involves mechanical hardware designs that automatically move resin vats, including cleaning and drying steps, as depicted in Fig. 5a. These systems are often tailored to meet the specific requirements of different materials. For instance, the tape-casting-integrated projection micro-stereolithography (PµSL) system was designed for MM 3d printing of the composite matrices with various fillers, where a doctor blade moves left to right to spread the material [108–110]. This process enables the production of lightweight, stiff, and high-damping composites by utilizing MM printing techniques with carbon fiber-reinforced polymers and elastic rubber.

Moreover, achieving fast and smooth transitions between different resin materials within the system is crucial for enhancing productivity [111, 116, 117]. Figure 5b illustrates a schematic diagram of MM-PµSL integrated with a dynamic fluidic control system, which enables rapid material exchange without interrupting the process [111]. Another approach involves a heterogeneous feedstock system based on in-situ resin mixing and exchange, allowing multiple resins to be used in a single vat, to produce metamaterials with diverse properties [10, 118, 119]. For precise printing, the removal of residual resin is also a critical step. Cheng et al. developed a centrifugal MM-DLP device that eliminates the need for separate resin residue cleaning as the printed green body moves between different material vats [120]. This system allows for non-contact, high-efficiency material switching using centrifugal force. Solution mask liquid lithography (SMaLL) technology allows printing without physical resin exchange. This method is characterized by its ability to operate without vat or resin exchange, instead using a wavelength-selective curing strategy, as shown in Fig. 5c. While the existing method relied on physical equipment control, this exciting technology uses chemistry in photopolymerization [112, 121].

MM 3D printing with functional materials offers enhanced control over the localized functionality of printed materials [32, 122]. Different composite materials and various additive manufacturing technologies can be combined to create functionalized materials tailored to meet diverse requirements, such as reversible activation [123], predictive deformation [124], and mechanical tunability [125]. For instance, electrically and magnetically dual-driven shape memory composites (SMCs) have been developed. Traditionally, SMCs have relied on a single source of stimuli; however, the ability to utilize multiple stimuli sources allows SMCs to operate more reliably, even under extreme conditions. Figure 5d shows that alternately printing high electric conductive and magnetic responsive layers enables simultaneous operation with Joule heat-based and high-frequency magnetic field induction-based stimuli [113]. Schwartz et al. developed a 4D printing material responsive to temperature stimuli and applied it as an actuator. They achieved mechanical anisotropy on the actuator, which controlled precise movements, and employed chemoselective wavelength-control methods to achieve MM actinic spatial control (MASC) [126]. Figure 5e shows another application where light-activated composites (LACs) that can modulate color patterns in response to light were fabricated. These LACs were created using a photoactive hydrogel composite consisting of polydopamine nanoparticles (PDA-NPs), acrylic acid hydrogel, and poly(ethylene glycol) diacrylate, and were printed using MM-based DLP 3D printing. These hydrogel composites can mimic cephalopods’ skin by changing the area of different colored regions in response to temperature variations [114]. Similarly, Joralmon et al. developed an MM-based DLP-printed object with unique optical properties that can reversibly transition from opaque (in the nematic phase) to transparent (in the isotropic phase) in response to external thermal stimuli using liquid crystals (LCs) [127].

MM 3D printing is gaining increasing attention as a next-generation technology for 3D electronics applications owing to its potential to produce lightweight, high-precision, and cost-effective products [128]. Zheng’s group demonstrated fabrication technology for 3D-patterned conductive and dielectric architectures with a wide range of mechanical stiffnesses and implemented an ultralightweight and deployable 3D multi-layer antenna system operating at an ultrahigh-frequency (19 GHz) [115]. Figure 5f illustrates the MM DLP technology used in this study, where negative and neutral resins are precisely printed using MM printing, allowing selective Cu plating only on the desired areas of the printed structure. Photocurable resins carrying tunable crosslink densities and charge polarities are placed at the electrode locations, enabling selective Cu plating on the printed structure later. In another study [129], they implemented a robotic metamaterial by combining photocurable resins with charge polarities and highly loaded ceramic nanoparticle colloids. This advanced technology implements a proprioceptive function, actively sensing and moving by interpenetrating piezoactive, structural, and conductive phases. Although photocuring-based printing has various advantages, it has limitations in introducing heterogeneous materials such as metals. However, these next-generation MM-based DLP technologies have demonstrated the possibility of integrating a diverse range of materials, such as metal electrodes, with additional processes.

### Grayscale DLP technology

**Fig. 6:**
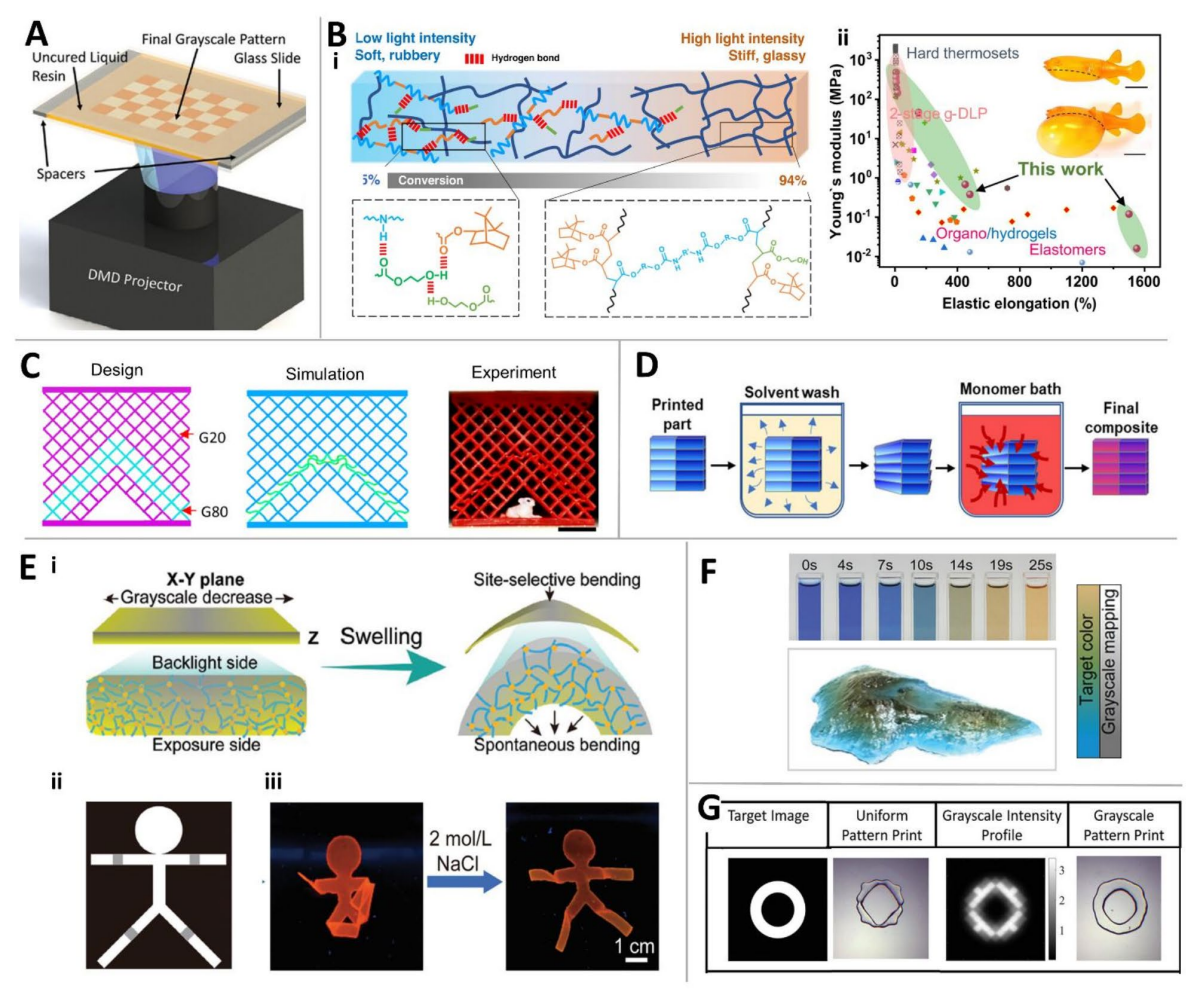
g-DLP technology and applications. (**a**) Schematic of g-DLP. Reproduced with permission from [134]. Copyright 2023, Wiley. (**b**) Achieving highly stretchable soft organogels to stiff thermosets within a single-vat printing. (i) Differentiated stretchability on the resin with different degrees of cure. (ii) The co-existence of stiff and elastomer regions with a modulus range from 0.016 to 478 MPa and stretchability up to 1500%. Reproduced with permission from [131]. Copyright 2023, Springer Nature. (**c**) Construction of highly FGMs using g-DLP. Reproduced with permission from [132]. Copyright 2019, AAAS. (**d**) Post-treatment approach for handling residual monomer in g-DLP. Reproduced with permission from [135]. Copyright 2022, Wiley. (**e**) (i) Principle of programming shape deformation of hydrogel using various swelling properties at different grayscales. (ii) Grayscale image design for specific shape deformation and (iii) results of achieving 3D shape deformation without asymmetric geometry or bi-layer configuration by utilizing nonuniform internal stresses. Reproduced with permission from [136]. Copyright 2022, Wiley. (**f**) Multicolor DLP printing via g-DLP by utilizing grayscale mapping to change color from blue to yellow. Reproduced with permission from [137]. Copyright 2022, Wiley. (**g**) Printing accuracy enhancement with g-DLP printing. Reproduced with permission from [134]. Copyright 2023, Wiley

Grayscale DLP (g-DLP) has emerged as a prospective strategy in additive manufacturing. In g-DLP printing, the local degree of monomer conversion is controlled by light intensity, which can be readily manipulated at the pixel level by the input grayscale image (Fig. 6a). Previously, to achieve gradient material properties within a single structure, MM printing had to be used. However, single-vat-based g-DLP provides a cost-effective solution that enables property variation within a single resin material [130]. In g-DLP 3D printing, the gel conversion rate is locally controlled through modulated light intensity. Accordingly, the physical and chemical properties of the material vary according to the spatially controlled crosslinking density. Although g-DLP uses a relatively simple mechanism of adjusting light intensity, it regulates chemical reactions and results in various secondary effects. This review introduces various research cases on g-DLP.

Qi’s research group demonstrated that using single-vat single-cure g-DLP, it is possible to achieve highly stretchable soft organogels to stiff thermosets within a single layer of printing [131]. They used three types of monomers: one type of stiff monomer and two types of soft monomers. They could achieve partially differentiated stretchability on the resin by using a mechanism where the stiffness of the stiff monomer increases according to the degree of cure, as shown in Fig. 6b-i. Additionally, at low conversion, residual monomers with hydrogen bonds act as solvent molecules in the organogel. They simultaneously co-existed in stiff and elastomer regions, as shown in Fig. 6b-ii. They demonstrated that the modulus could vary significantly from 0.016 to 478 MPa, with stretchability reaching up to 1500%.

The same research team also utilized g-DLP to construct highly functional gradient materials (FGMs) [132], such as complex 2D/3D lattices with controlled buckling and deformation sequences, negative Poisson’s ratio metamaterials, presurgical models with stiffness variations, composites for 4D printing, and anti-counterfeiting 3D printing [133]. Figure 6c shows examples of g-DLP printed FGMs with a mechanical gradient up to three orders of magnitude and high resolution, including lattice-based metamaterials. These were directly fabricated using grayscale light patterns and two-stage curing ink. A notable feature is that the two-stage curing hybrid ink system maximized the removal of residual monomer while maintaining the gradient of mechanical properties. This is a contrasting strategy to previous research [131], where residual monomer was left to achieve stretchability.

Addressing the challenge of increasing residual monomer levels as the degree of cure diminishes necessitates a tailored approach that considers the resin material’s characteristics and the research objectives. Hergert et al. presented an innovative study introducing a post-treatment methodology to address residual monomer concerns [135]. They employed spatial light intensity modulation with g-DLP to craft a 3D-printed polymer blend by expanding the resulting part with a secondary monomer. Figure 6d shows the resulting part swelling in a second monomer following g-DLP 3D printing. Through secondary polymerization, two distinct polymer networks can coexist within the structure. The controlled formation of these dual phases facilitates the removal of unreacted printing monomers and holds significant potential for achieving exceptional physical properties.

g-DLP technology offers a versatile approach for customizing the mechanical properties of 3D-printed objects and controlling their chemical attributes. Figure 6e-i and e-ii show the innovative application of g-DLP in implementing programmable shape deformation of hydrogels derived from a single precursor by manipulating their swelling properties through a grayscale pattern [136]. The concept of grayscale SLA designates the spatially varying crosslinking density of hydrogels, leading to distinct site-specific heterogeneity in physicochemical behavior, particularly the swelling capacity, as demonstrated in this study. The differential internal stresses within the printed hydrogel, induced by the non-uniform swelling properties, act as driving forces for the hydrogel to deform into the intended shape across three dimensions (Fig. 6e-iii). The gradient hydrogel with site-selective shape deformation demonstrates that targeted deformation is achievable through g-DLP without necessitating asymmetric geometry or bi-layer configuration [138].

In this way, g-DLP is an advantageous technology for motion programming with precise shape control. To precisely predict and control deformations, the finite element method (FEM) has been employed. Zhang et al. developed a design and fabrication framework for g-DLP-printed soft robots, inspired by the dexterous motions of natural organisms [139]. This framework combines a grayscale-dependent hyperelastic constitutive model and a voxel-based FEM approach. The constitutive model establishes the relationship between the projected grayscale values and the nonlinear mechanical properties, enabling the calculation of mechanical performance with arbitrarily distributed material properties [140]. Furthermore, this programming capability extends to shape memory polymer (SMP) technology, contributing to advancements in 4D printing. These techniques leverage the variations in thermomechanical properties enabled by g-DLP [77]. Hydrophilic UV curable shape memory polymers have been proposed for dual information encryption, offering superior mechanical properties by combining geometric shape memory and humidity-responsive transparency switching [141]. Additionally, g-dLP-printed polymers have been developed for use in cold-programming, eliminating the need for heat treatment in SMPs [87].

g-DLP presents a solution for directly introducing multiple colors with a single-vat, single-batch process, as illustrated in Fig. 6f [137]. Moreover, g-DLP can enhance the shape accuracy of 3D printing, as demonstrated by Montgomery et al. Figure 6g shows enhanced accuracy at the pixel level achieved using g-DLP printing [134]. In this approach, pixel-level grayscale manipulation with a reaction-diffusion modeling framework achieves high-resolution 3D printing that closely adheres to the target image [142, 143].

The significant advantage of g-DLP technology is its ability to integrate multiple properties while minimizing production time and energy consumption. However, g-DLP still faces challenges in maintaining the long-term stability of material properties. The properties produced by grayscale 3D printing may change over time, particularly when exposed to light (UV) or heat. Consequently, further research is needed to develop appropriate post-processing methods to preserve the intended functionality.

In g-DLP, it is crucial to develop techniques for evaluating the accuracy and precision of both the shape and the gradient, ensuring that the final print accurately reflects the intended design. For instance, a transition region of a few pixels can be observed between areas with different cured depths corresponding to varying grayscale levels. Furthermore, when there is a significant difference between adjacent grayscale levels, differences in curing kinetics can lead to variations in curing speed, potentially causing issues like overcuring due to the diffusion of free radicals [21]. This challenge is further complicated by the fact that the issue can vary depending on the resin material used in g-DLP, making it a critical area for ongoing research.

### Integrating AI with DLP 3D printing

3D printing integrates technologies from multiple fields, including material science, mechanics, and electronics. Consequently, AI can be leveraged for numerous purposes, ranging from material selection to enhancing the quality of the final product [144]. In material and structural design, AI has been used for design optimization, material development, and predicting mechanical properties. AI is employed for predicting shape deviations, detecting defects, and modeling processes to optimize the printing process [145–148].

When integrating AI into 3D printing, two critical aspects must be considered: (1) constructing a machine learning (ML) pipeline tailored to specific objectives and (2) acquiring high-fidelity data. The latter is fundamental for enhancing the performance of AI algorithms. However, in DLP 3D printing, the diversity of materials and parameters and their interactions during the photochemical reaction poses a significant challenge owing to the generation of sufficient high-fidelity data that accurately represents this complexity. Consequently, research on AI-driven DLP 3D printing has not advanced as extensively as in other 3D printing technologies, such as fused filament fabrication (FFF) and selective laser sintering [144]. This section highlights various efforts to integrate AI technology into DLP 3D printing.

**Fig. 7:**
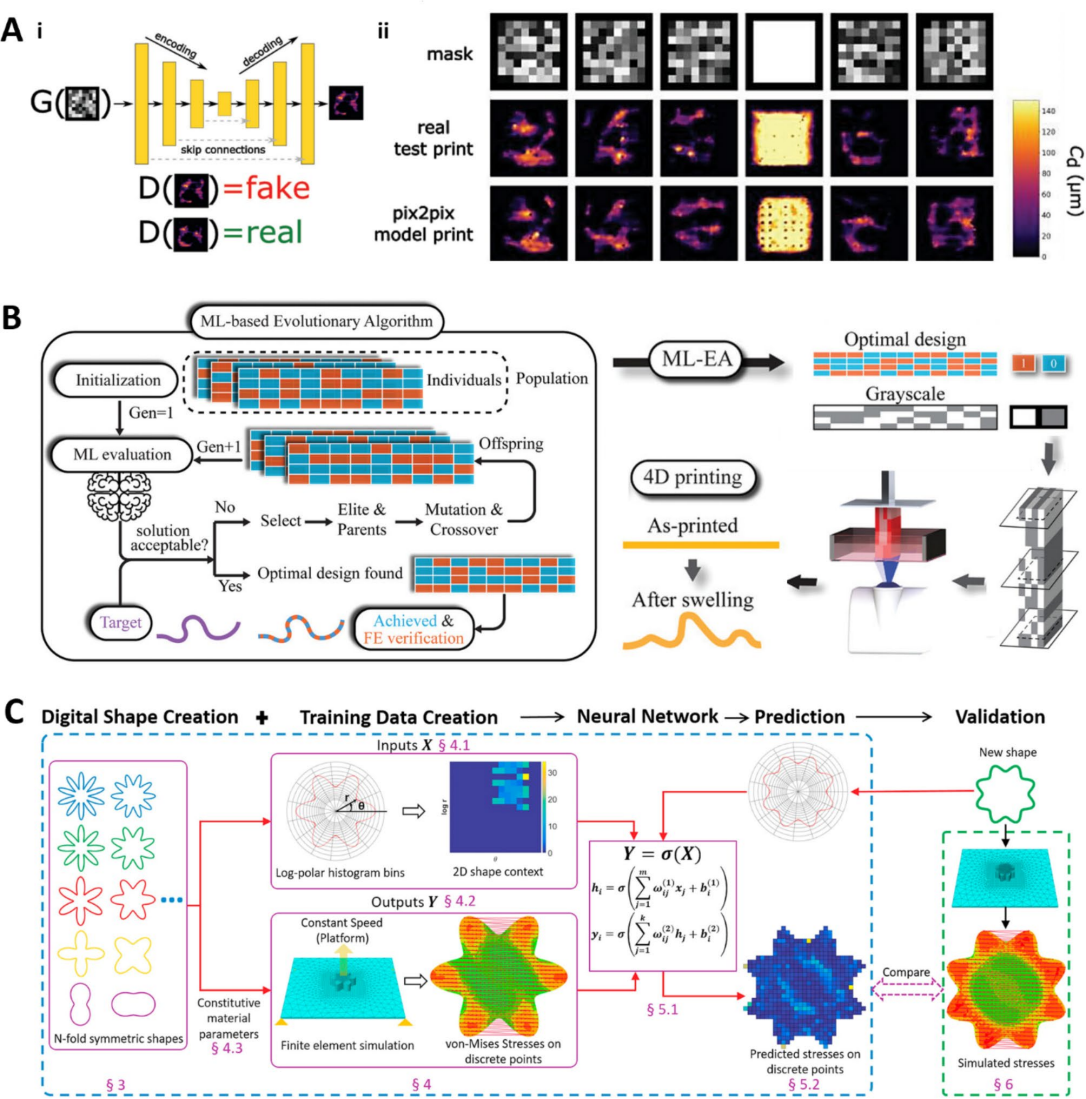
AI-driven DLP technologies. (**a**) (i) Pix2pix structure with U-Net-based encoder–decoder configured generator and cGAN-configured discriminator. (ii) Actual printed shape according to the mask image and the print shape predicted by the pix2pix architecture. Reproduced with permission from [150]. Copyright 2023, Wiley. (**b**) Grayscale design optimization via ML-based evolutionary algorithm (ML-EA) for actuation control in 4D printing. Reproduced with permission from [153]. Copyright 2022, Wiley. (**c**) Neural network (NN)-based ML model predicting stress distribution during the separation process after layer curing. Reproduced with permission from [156]. Copyright 2018, Elsevier

Image processing is the most actively pursued AI application strategy in DLP research for several reasons: (1) Image data are inherently produced in DLP because of the use of photomask-based layer-by-layer printing. (2) The mask image, by locally adjusting light energy, determines the material properties at specific locations. DLP relies on a set of sliced pixel-based grayscale images as input, as mentioned in Sect. 2. Therefore, research has been conducted to improve printing accuracy by precisely adjusting shapes to mitigate the effects of light scattering. In this process, AI-based image processing technology has been employed to derive optimized printing mask designs for various structures [144, 149]. With the advancement of g-DLP technology, adjusting the brightness values of each pixel can produce advanced materials, as discussed in Sect. 4.2. A dataset can demonstrate that grayscale images are easier to handle than RGB images because each pixel has a one-dimensional input value. This simplification enables the image to be converted into a 2D matrix. In specific cases, U-Net-based ML models, including the pix2pix conditional generative adversarial network (cGAN), have been employed to optimize black-and-white digital masks, thereby achieving improved printing accuracy [150]. Figure 7a-i illustrates the pix2pix structure, which includes a U-Net-based encoder–decoder configured generator and a cGAN-configured discriminator. Figure 7a-ii shows the actual printed shape based on the mask image and the print shape predicted by the pix2pix architecture.

Studies have been conducted to predict the mechanical behavior of structures based on the grayscale pattern. Grayscale images can control the mechanical properties of photocurable materials, as addressed in Sect. 4.2. By leveraging this capability, grayscale pattern blocks optimized for the desired deformation were derived [150]. This controllability of the printed structure’s behavior also opens the possibility for AI-based 4D printing applications [151, 152]. For instance, Sun et al. demonstrated a technique whereby an ML-EA was applied to adjust the shape as desired during actuation [153]. Figure 7b illustrates the process of forming an optimal grayscale patterned block using ML-EA and its application in controlling 4D printing.

AI technology is increasingly being implemented to enhance the performance of materials in DLP. Therefore, material development and process optimization are considered simultaneously [154]. Because the mutual relationship between materials and processes is a strategic approach, DLP printing significantly influences the physical properties of materials from resin preparation to post-processing. For example, a method for producing density-controlled alumina ceramics via DLP using ML technology has been proposed [155]. In this method, an NN predicts shrinkage and porosity, enabling the creation of dense and porous ceramic cross-structures. The final product is predicted using material composition, printing parameters and post-processing steps as input data.

Additionally, efforts are underway to enhance DLP printing performance further. Wang et al. embedded an NN-based ML model to predict the stress distribution exerted on structures during the separation process after layer curing, as illustrated in Fig. 7c [156]. This approach enables the early identification of potential structural weaknesses during the printing process.

The development of in-situ monitoring systems represents a promising future direction for AI-DLP technology [157]. By incorporating Internet of Things (IoT) technology into the DLP process, real-time monitoring and control systems could be developed, similar to the G-code-based real-time control used in FFF [144]. Such innovations would enable rapid detection of printing process failures, enhance product quality, and reduce material waste.

### Other DLP innovations

**Fig. 8:**
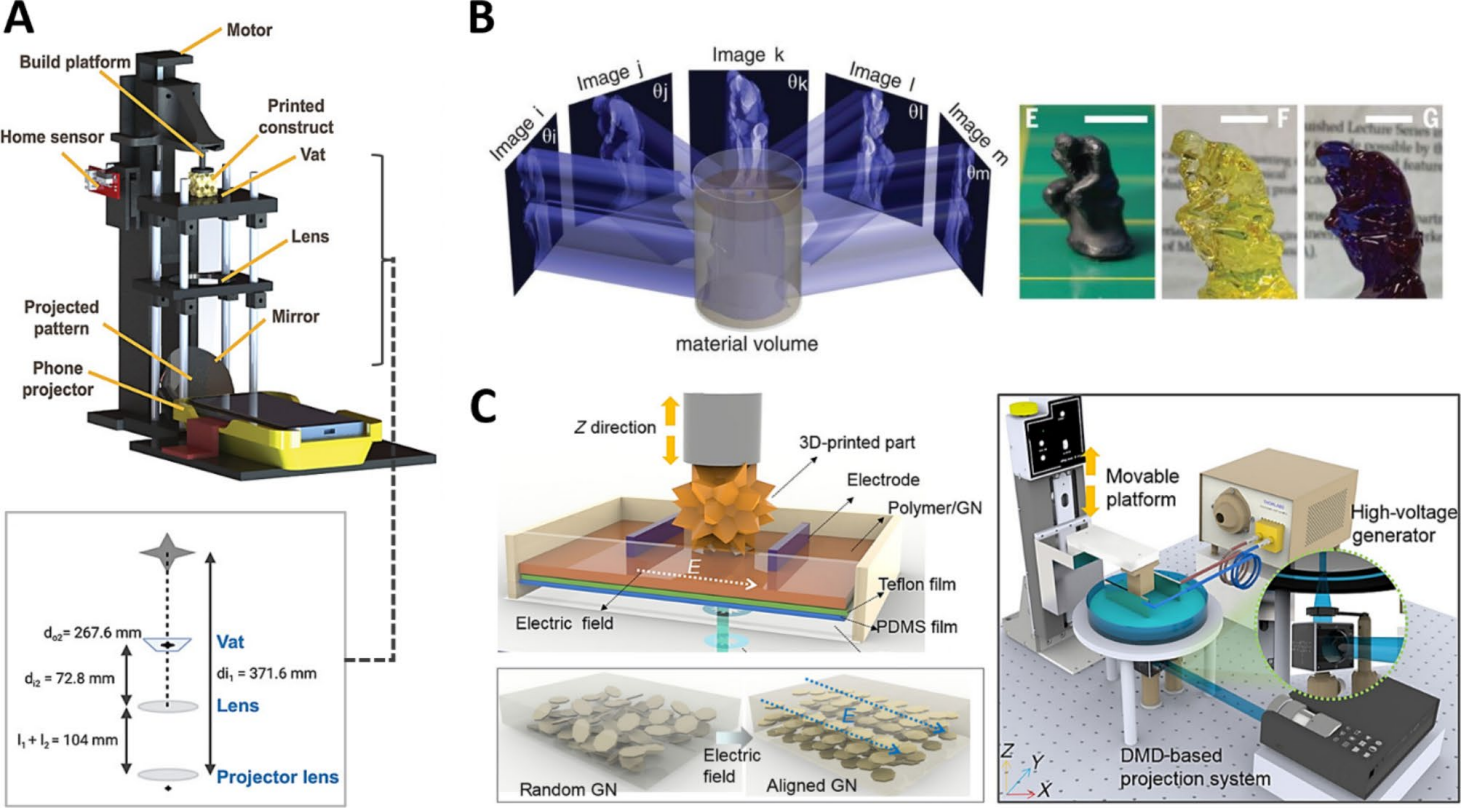
Advancements for improving the usability and scalability of DLP equipment. (**a**) Schematic diagram of smartphone-based DLP equipment. Reproduced with permission from [158]. Copyright 2021, Wiley. (**b**) (i) Volumetric DLP creating patterned light fields in 3D space with multiple light beams and (ii) printed objects in high resolution. Reproduced with permission from [159]. Copyright 2019, AAAS. (**c**) Electrically assisted DLP technology using electrodes to apply voltage to the resin. It enables precise control of the microstructure of materials and the schematic diagram of the full system. Reproduced with permission from [162]. Copyright 2019, AAAS

In addition to the previously reviewed technologies, several advancements are being made to enhance the usability and scalability of DLP equipment [5]. For instance, a study investigated the implementation of portable DLP technology using smartphones. A portable 3D DLP printer was developed using a smartphone-powered projector and a custom-designed smartphone-operated app. Figure 8a illustrates a schematic diagram of this smartphone-based DLP equipment. This technology has demonstrated potential contributions to mobile health technology owing to its portability [158].

Volumetric DLP is an emerging technology with significant potential for advancing the next generation of DLP systems. Traditional DLP printing operates through a layer-by-layer process using a single planar light beam. In contrast, volumetric DLP generates patterned light fields in three-dimensional space using multiple light beams, as shown in Fig. 8b. This method offers advantages by eliminating the physical stress typically applied during the peeling step of the curing process and by overcoming structural form constraints [159]. Furthermore, this volumetric stereolithography has been extended to bioprinting and successfully utilized to print trabecular bone models [160]. Recently, xolography, a variant of volumetric DLP technology, has also advanced by improving accuracy. The use of continuously flowing resin minimizes unwanted polymerization, thereby improving precision and increasing the potential for continuous production through parallel printing. This approach has been reported to resolve the trade-off between production speed and resolution [161].

Other techniques have been proposed to enhance the productivity of DLP manufacturing. Walker et al. introduced a new 3D printing method called high-area rapid printing (HARP), which utilizes a mobile liquid interface (fluorinated oil) to minimize adhesive forces between the interface and the printed object, thereby enabling high-speed printing [163]. This technology reportedly achieves vertical print speeds exceeding 430 mm per hour and a throughput of 100 L per hour with various materials. Electrically assisted DLP is also a strategy of DLP 3D printing. This approach uses electrodes to apply voltage to the resin in the vat, enabling precise control over the microstructure of materials, as shown in Fig. 8c. An electric field is generated to align nanoparticles under direct current (DC) voltage efficiently. By adjusting and rotating the electrodes, the organizational structure of the nanoparticles can be finely tuned, mimicking the excellent structural characteristics found in natural composites [162]. Additionally, there are notable examples of equipment that integrates multiple 3D printing technologies. Peng et al. introduced a novel hybrid multi-material 3D printing system that combines top-down DLP with DIW to produce composite structures and distinctive devices within a single printing operation [164]. While the integration of DLP-based printing technology is beneficial for incorporating a variety of materials, combining it with other existing 3D printing methods [165–167] that are optimized for specific materials represents a particularly advantageous strategy for material fabrication.

## Challenges and future directions

This review highlights the innovative materials and system advancements in DLP 3D printing, a prominent technique within vat photopolymerization-based additive manufacturing. Following the Introduction in Sect. 1, Sect. 2 presents the fundamentals of DLP, outlining key processing parameters and performance metrics intrinsic to the technology. Section 3.1 addresses recent innovations in materials, with a particular emphasis on the development of functional materials such as piezoelectric composites and ceramics suitable for DLP applications. The 3D printing of piezoelectric materials is particularly significant due to the potential integration of intrinsic material properties, such as piezoelectricity, with structural characteristics unique to additive manufacturing. This combination enables new applications previously unattainable, including real-time programmability and advanced signal processing. Despite the growing interest in DLP technologies for fabricating piezoelectric ceramics and ceramic composites—key components in this field—current advancements have not kept pace with those observed in polymers. Therefore, this review specifically focuses on the reported DLP techniques for piezoelectric ceramics and composites. Furthermore, in the later part of Sect. 3.1, we examine the current state of 4D printing enabled by DLP technology, which incorporates responsive materials such as shape memory polymers and hydrogels. We discuss the technological advancements achieved to date in this area and outline directions for future research and development. Additionally, in Sect. 3.2, we discuss emerging trends in the development of recyclable resin materials for sustainable digital light processing (DLP) applications. Recyclable DLP technology is essential for achieving sustainable and environmentally friendly manufacturing. However, a review of current findings indicates that the reuse of certain photocurable resins is constrained, primarily due to reliance on specific recycling methods and a limited selection of bio-based materials. Consequently, extracting recycled resins that exhibit performance comparable to pristine materials presents a significant challenge.

**Fig. 9:**
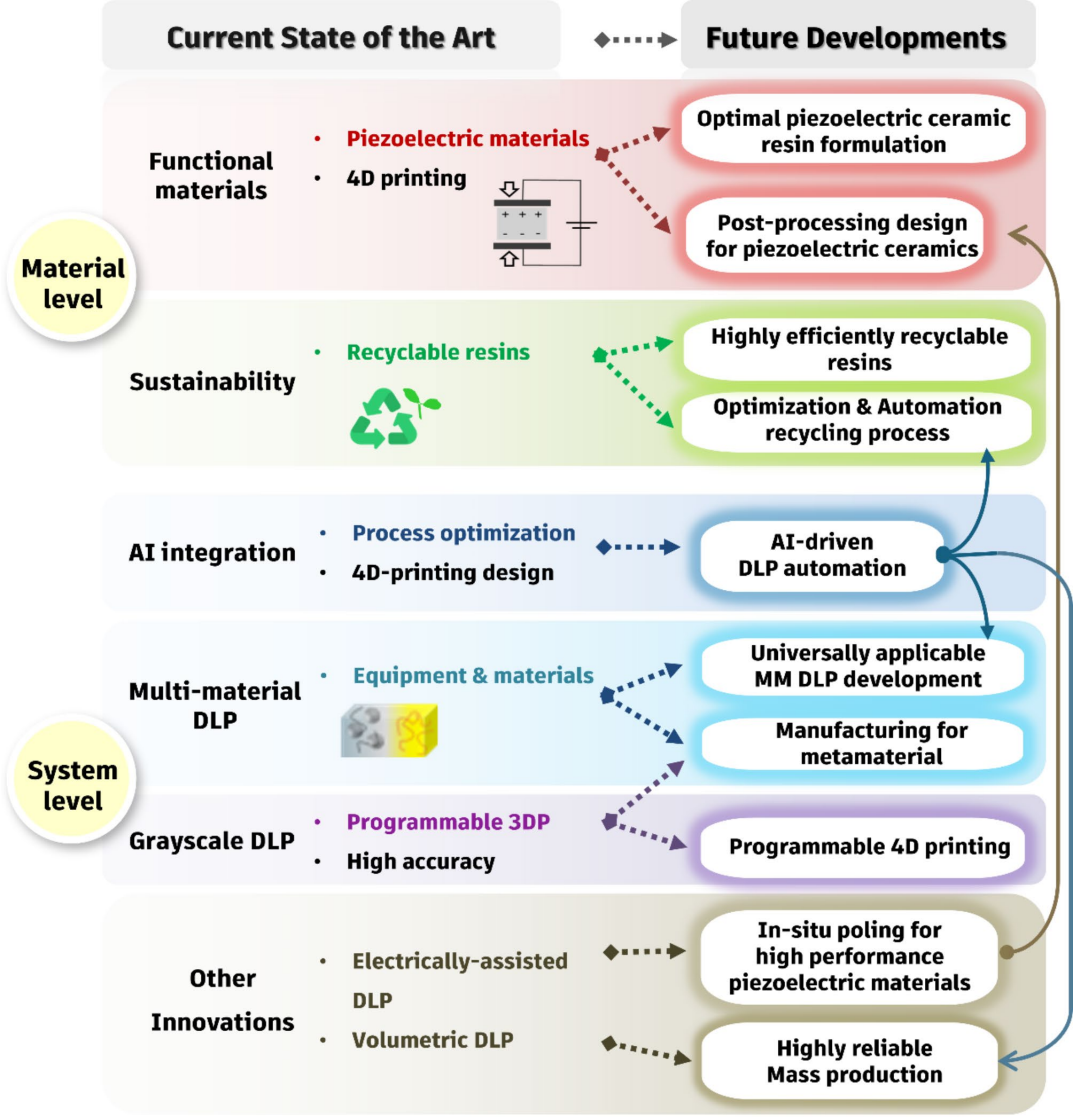
State-of-the-art and future directions of DLP. The future possibilities in both material innovations—such as recyclable resins and piezoelectric materials—and system-level advancements, including AI integration and automation

In addition, we address innovations in DLP systems designed to meet various objectives. As discussed in Sect. 4.1, the realization of multi-material capabilities within a single 3D-printed architecture requires significant advancements in equipment. While several promising multi-material 3D printing systems have been proposed, they are not yet universally applicable and currently accommodate only specific material combinations. Furthermore, these systems are predominantly confined to lab-scale applications. Therefore, there is a pressing need for the development of more universally applicable multi-material 3D printing solutions that incorporate automation. In Sect. 4.2, we emphasize the potential of g-DLP, which offers new opportunities for tailoring the mechanical properties of programmable 3D-printed structures and enhancing dimensional accuracy. Although this area remains in its early stages, notable advancements have been made. Section 4.3 provides an overview of the integration of AI within DLP 3D printing. The application of AI in DLP is still relatively limited, highlighting a significant area for future exploration. Lastly, Sect. 4.4 reviews various DLP innovations, including applications utilizing smartphones and volumetric DLP technologies. In summary, despite significant progress in both materials and technologies that enhance the capabilities of DLP, various challenges persist due to the relatively nascent state of this technology. This presents numerous opportunities for further research and innovation. Figure 9 illustrates the state-of-the-art advancements and future directions of DLP 3D printing, highlighting both material innovations and system developments. It outlines the potential for further breakthroughs in areas which are expected to drive the next generation of DLP technologies.

First, from a materials perspective, advancements in ceramic digital light processing (DLP) technology, particularly for piezoelectric ceramics, are less developed compared to those for polymers and metals. The ceramic DLP process necessitates the mixing of ceramics with conventional resins and the formulation of resins that minimize the refractive index differences between the ceramics and the photocurable resin. This minimization is crucial for promoting efficient light absorption during curing while maintaining low viscosity during the mixing of ceramics. For green bodies produced by DLP 3D printing, the ceramic structures are embedded within a cured polymer matrix. Consequently, the design of post-processing steps—such as de-binding and densification through sintering—is essential for effectively removing the polymer resin while preserving the integrity of the target ceramic structure. Proposed strategies for enhancing this process include designing a multi-step thermal profile or conducting de-binding and sintering under controlled atmospheres. Traditionally, high pressure is applied during non-3D printing methods to promote densification of ceramics. However, this pressure cannot be applied to 3D-printed ceramic bodies, necessitating innovative process approaches that can replicate the densification effects typically achieved through pressure. Additionally, for piezoelectric ceramics, achieving the desired properties through DLP 3D printing requires complex post-processing steps, including poling for polarization. The development of equipment capable of applying sufficient electric field in real-time during the DLP process to align dipoles will be crucial in addressing these challenges.

Second, with respect to the recycling of DLP resin materials, significant challenges persist despite several closed-loop DLP recycling processes that have been proposed. Notably, these challenges include the performance degradation of recycled resins relative to their original state and the necessity of replenishing specific chemicals during the reuse process. Research into additive manufacturing technologies focused on sustainability is essential and should be pursued continuously in a manner that is more environmentally friendly than existing methods. However, many of these technologies suffer from reduced efficiency and limitations in material selection. To address these issues, it is imperative to develop more efficient formulations for recycled resins as well as advanced equipment systems. Particularly, it is crucial to establish a family of resins that not only facilitates efficient recycling processes but also maintains high performance, rather than merely reusing widely employed resins. In sustainable DLP 3D printing, AI algorithms show considerable promise for minimizing material usage and optimizing material reuse by reintroducing unused material back into the vat. Utilizing AI algorithms to reduce unnecessary material consumption in DLP processes represents a viable solution that aligns well with sustainability goals, similar to applications already implemented in other 3D printing processes.

Third, advancing MM DLP technology necessitates the development of universal printing methods and equipment capable of simultaneously printing multiple materials. This advancement is particularly crucial for the fabrication of electronic components, where the formation of electrodes alongside functional elements that possess dielectric, ferroelectric, or piezoelectric properties is essential. However, the challenge of achieving simultaneous 3D printing of conductive and insulating materials in a single vat remains a significant hurdle. While the advancements reported to date in multi-material DLP systems are impressive, they typically allow for the combination of only specific materials, limiting broader applications. The ability to universally combine a diverse array of materials would not only enhance the versatility of DLP technology but also expand its potential applications across various fields. Additionally, the significance of DLP 3D printing extends to the development of mechanical metamaterials, which are engineered materials with unique mechanical properties not found in nature. The precise layering and material combination capabilities of DLP printing enable the fabrication of complex structures that exhibit tailored stiffness, elasticity, and other mechanical behaviors, paving the way for innovative designs in lightweight and responsive materials [168–171]. Furthermore, wave-based metamaterials, which can manipulate wave propagation—such as sound, electromagnetic waves, or seismic waves—also stand to benefit from advances in DLP technology. By utilizing DLP’s precision and multi-material capabilities, researchers can create sophisticated devices that offer unprecedented control over wave phenomena, leading to applications in acoustics, optics, and beyond [172–176]. Moreover, extending g-DLP to encompass a wider range of materials may facilitate the development of programmable 4D printing structures, allowing for the integration of unique structural designs and dynamic functionalities. By incorporating materials that respond to environmental stimuli, future MM DLP systems could enable the creation of sophisticated components with adaptive capabilities, further paving the way for innovations in sectors such as electronics, biomedical devices, and mechanical metamaterials [177, 178]. Overall, addressing these challenges will be instrumental in unlocking the full potential of MM DLP technology and g-DLP, driving advancements in manufacturing processes and fostering new applications in diverse industries.

Lastly, the development of mass production systems with robust quality control capabilities is essential for DLP to become a viable option for large-scale manufacturing. To facilitate this transition, it is imperative to enhance printing speeds while establishing effective quality control systems capable of monitoring and maintaining the integrity of each printed part throughout the production process. Additionally, there is a critical need to formulate scalable resins that not only meet the performance requirements for various applications but also allow for efficient recycling and sustainability. Moreover, the automation of post-processing procedures—including washing, curing, and finishing—will further increase production efficiency and reduce turnaround times. Incorporating advanced technologies, such as artificial intelligence and machine learning, can optimize printing and post-processing parameters, leading to improved overall performance and reduced material waste. Addressing these challenges will not only improve the operational efficiency of DLP but also significantly unlock its untapped potential, thereby positioning it as a leading technology in the realm of additive manufacturing. As the demand for high-quality, customized products continues to grow across various industries, the ability of DLP to meet these needs through scalable and efficient production processes will be a critical factor in its future success.

In conclusion, as we address these technological and material challenges, in the future, DLP 3D printing will be not only more efficient and versatile, but also more sustainable and scalable. This evolution will open new avenues for applications across various industries, fostering innovation and advancing the frontiers of additive manufacturing. Continued interdisciplinary collaboration and research will be crucial in driving these advancements and realizing the full potential of DLP technology.

## Data Availability

Not applicable.
